# Capacity for and Utilization of Dual‐Energy X‐Ray Absorptiometry Within the Veterans Health Administration

**DOI:** 10.1111/jgs.70222

**Published:** 2025-12-05

**Authors:** Heather Davila, Kimberly D. McCoy, Michelle A. Mengeling, Radhika R. Narla, Melissa J. A. Steffen, Karla L. Miller, Samantha L. Solimeo

**Affiliations:** ^1^ VHA Office of Rural Health, Veterans Rural Health Resource Center‐Iowa City, Iowa City VA Health Care System Iowa City Iowa USA; ^2^ Center for Access & Delivery Research and Evaluation, Iowa City VA Health Care System Iowa City Iowa USA; ^3^ Department of Internal Medicine University of Iowa Carver College of Medicine Iowa City Iowa USA; ^4^ Division of Endocrinology, Metabolism, and Nutrition VA Puget Sound Health Care System Seattle Washington USA; ^5^ Division of Metabolism, Endocrinology and Nutrition, Department of Medicine University of Washington School of Medicine Seattle Washington USA; ^6^ Primary Care and Rheumatology Section VA Salt Lake City Healthcare System Salt Lake City Utah USA; ^7^ Division of Rheumatology University of Utah School of Medicine Salt Lake City Utah USA

**Keywords:** access, fracture risk assessment, veterans

## Abstract

DXA Utilization Among Veterans Aged ≥ 50 years by Facility‐Reported DXA Capacity.

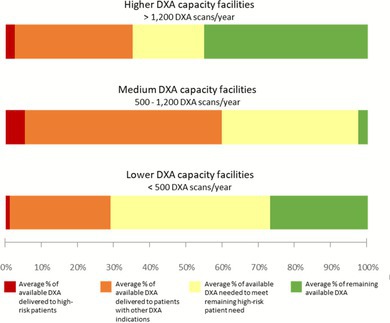

## 1 Introduction

Approximately 50% of women and 25% of men aged ≥ 50 years have osteoporosis, a condition characterized by reduced bone mass and compromised bone architecture, leading to a higher risk of fractures [1]. Fractures among older adults are common and associated with significant morbidity and mortality [2]. By 2040, the number of fractures in the United States is expected to increase by 68% to reach 3.2 million annually, with costs over $95 billion [2]. This burden could be reduced with targeted screening and treatment.

Bone mineral density (BMD) is a crucial indicator of fracture risk; guidelines recommend screening those at high fracture risk using dual‐energy x‐ray absorptiometry (DXA) to measure BMD and diagnose osteoporosis [1]. Despite its availability, DXA imaging remains underutilized for patients at risk for first fracture and those with prior fracture indicating high risk for recurrence [3, 4]. Hip fracture rates increased among male Veterans aged ≥ 50 years between 2006 and 2019, although fewer than 6% underwent DXA imaging within 2 years post‐fracture [5]. A recent clinical trial involving male Veterans aged 65 to 85 years found DXA rates of 3.2% in the control group [6].

The Veterans Health Administration (VHA) is the largest integrated healthcare system in the United States, providing healthcare to 9 million Veterans, with emphasis on primary care and preventive services. Our goal was to evaluate VHA's internal capacity to meet the demand for DXA imaging among Veterans at high fracture risk.

## 2 Methods

In 2021, a survey was administered to a national sample of VHA facilities to assess VHA's current state of DXA imaging; 56 facilities (31%) participated [7]. This study represents a secondary analysis of data derived from the survey and VHA's Corporate Data Warehouse, a national repository of patient and facility data. The study was reviewed by the University of Iowa Institutional Review Board and received a non‐research determination.

We assessed DXA capacity using two survey items. The first item asked for the number of weekly DXA appointments available at the facility. The second item asked if walk‐in DXA scans were provided. We estimated annual DXA capacity for each facility by multiplying the number of weekly appointments by 48 weeks (vs. 52 weeks) to account for events that could affect DXA availability. For sites that only offered walk‐in appointments (*n* = 4), we estimated 40 available appointments/week based on the mean number of walk‐in appointments at sites reporting this information. We did not include walk‐in appointments in our estimates for sites that offered both scheduled and walk‐in visits.

To assess utilization, we created a cohort of patients associated with participating facilities based on DXA receipt at the facility or nearest primary care clinic. We included patients aged 50–99 years with ≥ 1 outpatient visit during fiscal years 2019–2020 (10/1/18–9/30/20). Among this cohort, we identified patients at high fracture risk based on recent diagnosis of Parkinson's disease, androgen deprivation therapy or orchiectomy, steroid use, aromatase use, or prior hip fracture [8]. We used procedure codes to assess DXA use during fiscal years 2021–2022 among the overall cohort, and among high‐risk patients.

## 3 Results

After excluding facilities with missing data for key variables, the sample included 46 facilities (Table 1). Among patients associated with these facilities (~1.5 million), about 1% were classified as high‐risk. During the two‐year follow‐up, 10.8% of high‐risk patients received DXA.

**TABLE 1:** Facility characteristics by self‐reported DXA capacity: Fiscal Years 2021–2022.

	Higher DXA capacity facilities	Medium DXA capacity facilities	Lower DXA capacity facilities	Overall
	N	N	N	
Total facilities	18	16	12	46
Patients per facility, mean (SD)	42,233 (19,908)	34,967 (22,241)	15,463 (6296)	32,722 (21,017)
Range	11,025–78,684	10,458–95,210	8367–25,852	8367–95,210
Patients who received DXA by facility, mean (SD)	636.4 (380.2)	547.6 (443.0)	96.9 (74.1)	464.8 (413.3)
Range	30–1263	17–1709	17–209	17–1709
High‐risk patients per facility, mean (SD)	434.6 (226.8)	383.6 (204.8)	137.3 (58.2)	339.3 (222.4)
Range	98–935	124–836	71–237	71–935
High‐risk patients who received DXA, mean (SD)	49.1 (31.3)	46.8 (33.4)	3.8 (3.9)	36.5 (33.6)
Range	3–100	0–104	0–10	0–104
High‐risk patients who received DXA, mean percent	11.3	12.2	2.8	10.8

*Note*: Fiscal years (FY) 2021–2022 = 10/1/20–9/30/22. Patients included in the analysis were ages 50–99 with ≥ 1 outpatient visit in VHA during FY2019‐2020. If a patient received DXA at a participating facility during FY2021–2022, they were associated with that facility. Other patients were assigned to VHA facility based on nearest primary care location. If a patient received ≥ 1 DXA during the study period, we used the date of the first DXA. Higher DXA capacity facilities = facility‐reported capacity to deliver > 1200 DXA scans/year; medium DXA capacity facilities = 500–1200 scans/year; lower DXA capacity facilities < 500 scans/year.

**FIGURE 1:**
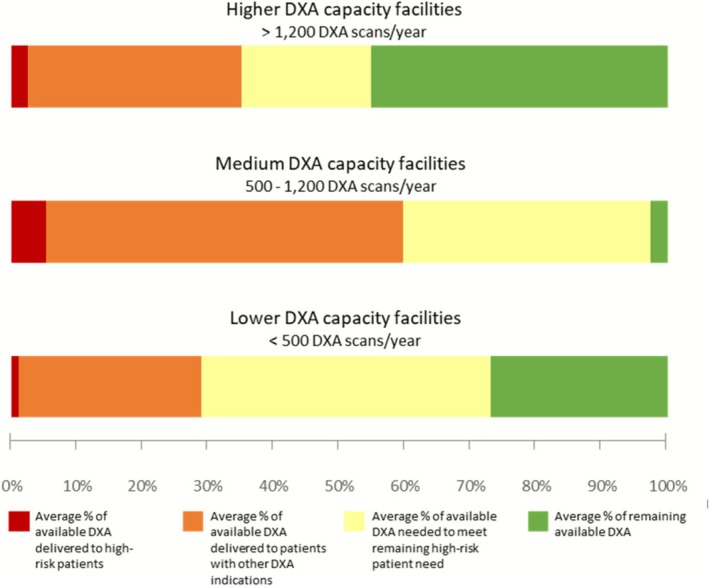
This figure shows the use of dual‐energy x‐ray absorptiometry (DXA) among Veterans aged ≥ 50 years at 46 Veterans Health Administration (VHA) medical centers. Facilities were grouped into three categories based on the number of DXA scans they reported having capacity to conduct each year: > 1200 DXA scans/year (“higher capacity”), 500–1200 DXA scans/year (“medium capacity”), and < 500 DXA scans/year (“lower capacity”). The stacked bar chart shows that, on average, facilities delivered a small proportion of their available DXA to patients at high‐risk of fracture (red bar). Even when adding those DXA scans delivered to patients with other DXA indications (orange bar), facilities reported sufficient capacity to meet the DXA needs of all high‐risk patients (yellow and green bars). DXA utilization by facility‐reported DXA capacity.

A small proportion of available DXA was utilized by high‐risk patients, although sites reported adequate capacity to meet high‐risk patients' DXA needs, even after accounting for DXA utilized by other patients (Figure 1). Those facilities reporting capacity to deliver 500–1200 DXA per year (“medium capacity”) used a higher percentage of their available DXA appointments overall and for high‐risk patients.

## 4 Discussion

Despite the low proportion of high‐risk Veterans who received DXA during the study, this analysis suggests limited DXA capacity within VHA is not the primary barrier. Consistent with previous studies [4, 5, 6], our finding of low DXA utilization highlights the need for education and outreach to patients and clinicians about the value of DXA in fracture risk reduction [6]. Further, access barriers, such as transportation challenges, may lead Veterans to delay or decline care. The expanded VHA‐financed care options under the 2018 MISSION Act may help address access gaps, particularly for Veterans living in rural areas [9].

Limitations of this analysis include our use of facility‐reported data to assess DXA capacity and low survey response rate. Subsequent research could examine the role of VHA‐financed community care in improving DXA access, as well as patient and clinician perspectives on strategies to improve DXA uptake. Regardless of where high‐risk Veterans receive care, our findings highlight an opportunity to improve DXA access, ultimately enhancing long‐term outcomes for those at risk of fracture.

## Author Contributions

All significant contributors to this work are listed as authors. All authors have reviewed and approved the final submission. **Heather Davila:** conception and design, analysis and interpretation of data, drafting manuscript, critical revision, final approval. **Kimberly D. McCoy:** acquisition of data, analysis and interpretation of data, drafting manuscript, critical revision, final approval. **Michelle A. Mengeling:** conception and design, acquisition of data, analysis and interpretation of data, drafting manuscript, critical revision, final approval. **Radhika R. Narla:** analysis and interpretation of data, drafting manuscript, critical revision, final approval. **Melissa J. A. Steffen:** acquisition of data, critical revision, final approval. **Karla L. Miller:** conception and design, acquisition of data, analysis and interpretation of data, critical revision, final approval. **Samantha L. Solimeo:** conception and design, acquisition of data, analysis and interpretation of data, drafting manuscript, critical revision, final approval.

## Funding

This material is based upon work supported by the Department of Veterans Affairs, Veterans Health Administration, Office of Rural Health, Veterans Rural Health Resource Center–Iowa City (Award # 03856).

## Disclosure

All authors are employees of the US Department of Veterans Affairs—Veterans Health Administration (VHA). The VHA had no role in the analysis or interpretation of data or the decision to report these data in a peer‐reviewed journal. The views expressed in this manuscript are those of the authors and do not necessarily reflect the position or policy of the VHA or the United States government.

## Conflicts of Interest

The authors declare no conflicts of interest.
